# Predictive validity of pre-admission assessments on medical student performance

**DOI:** 10.5116/ijme.5a10.04e1

**Published:** 2017-11-24

**Authors:** Al-Awwab Dabaliz, Samy Kaadan, M. Marwan Dabbagh, Abdulaziz Barakat, Mohammad Abrar Shareef, Mohamad Al-Tannir, Akef Obeidat, Ayman Mohamed

**Affiliations:** 1 College of Medicine, Alfaisal University, Riyadh, Kingdom of Saudi Arabia; 2 Mercy St. Vincent Medical Center, Toledo, United States; 3 King Fahad Medical City, Riyadh, Kingdom of Saudi Arabia

**Keywords:** Standardized admission tests, predictive validity, college performance indicators, cumulative grade point average, progress test

## Abstract

**Objectives:**

To examine the predictive validity of pre-admission variables on students’ performance in a medical school in Saudi Arabia.

**Methods:**

In this retrospective study, we collected admission and college performance data for 737 students in preclinical and clinical years. Data included high school scores and other standardized test scores, such as those of the National Achievement Test and the General Aptitude Test. Additionally, we included the scores of the Test of English as a Foreign Language (TOEFL) and the International English Language Testing System (IELTS) exams. Those datasets were then compared with college performance indicators, namely the cumulative Grade Point Average (cGPA) and progress test, using multivariate linear regression analysis.

**Results:**

In preclinical years, both the National Achievement Test (p=0.04, B=0.08) and TOEFL (p=0.017, B=0.01) scores were positive predictors of cGPA, whereas the General Aptitude Test (p=0.048, B=-0.05) negatively predicted cGPA. Moreover, none of the pre-admission variables were predictive of progress test performance in the same group. On the other hand, none of the pre-admission variables were predictive of cGPA in clinical years. Overall, cGPA strongly predict-ed students’ progress test performance (p<0.001 and B=19.02).

**Conclusions:**

Only the National Achievement Test and TOEFL significantly predicted performance in preclinical years. However, these variables do not predict progress test performance, meaning that they do not predict the functional knowledge reflected in the progress test. We report various strengths and deficiencies in the current medical college admission criteria, and call for employing more sensitive and valid ones that predict student performance and functional knowledge, especially in the clinical years.

## Introduction

The medical school admission process is an ever-growing topic on which the literature is voluminous. Scrutiny and filtration of student applicants have become more meticulous than ever before due to the increasing number of applicants, the increasing proportion of outstanding ones, and the continuous strive for maintaining the highest possible standard of students. Studies have questioned the predictive validity of the various pre-admission variables on college academic performance. The necessity of further validation and refinement of pre-admission variables that are invalid or weakly predictive has been established in several studies.^1^^-^^3^

In Saudi Arabia, medical schools depend on a composite of pre-admission variables in their student selection. The main pre-admission variables currently being used are the cumulative high school average, the National Achievement Test score (NAT, locally known as Tahsili), the General Aptitude Test score (GAT, locally known as Qudrat), in addition to the English language proficiency exam score.

Conventionally, pre-admission assessments aim to cover academic and non-academic cognitive variables in addition to English language proficiency. Firstly, academic achievement assessment tools cover some forms of cognitive skills, mainly the student's knowledge base.^4^ According to the literature, this category is believed to be the most reliable in predicting academic performance in medical school.^5^^-^^10^ Such tools include high school averages, Medical College Admission Test (MCAT), A-levels, and SAT Subject Tests. In Saudi Arabia, the NAT is used in assessing the cumulative knowledge acquired throughout the final three years of high school in five subjects: biology, chemistry, physics, mathematics and English.^11^^, ^^12^

Secondly, there is a general literature agreement in regards to the significance of cognitive non-academic assessment tools in selecting medical students.^13^^-^^16^ These cover a wide range of competencies that are essential for success in the field of medicine, particularly in the clinical years, such as problem-solving, critical reasoning and quantitative skills.^10^^,^^17^ Their predictive validity, however, is uncertain.^1^^-^^3^ Examples of such tools include the United Kingdom Clinical Aptitude Test (UKCAT) and the Undergraduate Medicine and Health Sciences Admission Test (UMAT). In Saudi Arabia, non-academic assessment is achieved through the GAT, which is composed of two sections: verbal and quantitative. The GAT aims to assess reading and comprehension abilities, understanding logical relationships, problem-solving skills based on mathematical concepts and culturally acquired knowledge.^18^

English has become the language of choice at many international colleges, the written language of most of the top leading journals and medical publications^19^ and of course international conferences. Without a fundamental command of the English language, students would most likely not be able to fulfill their assignments or study goals and thus not be able to attain their maximal performance potential as the lack of adequate language competence could act as a major limiting factor. Tools that assess English language proficiency cover various aspects of the language domain, including reading comprehension, writing, listening, and speaking. Several studies have shown that English language proficiency affects academic performance among non-natives as with the case in Saudi Arabia and other Middle Eastern countries.^20^^-^^23^ Moreover, this has also been a concern among natives in medical schools of English speaking countries such as Australia, as reported by Hayes and colleagues.^20^^-^^23^ Two of the most widely used assessment tools for English proficiency are the Test of English as a Foreign Language (TOEFL) and the International English Language Testing System (IELTS). These are now standard exams used and recognized worldwide by thousands of institutions.^24^^, ^^25^ Despite their popularity, these exams have not been used in predicting medical school performance in previous studies.

Predictive validity studies commonly use college performance indicators in examining pre-admission variables.^6^^, ^^26^ A widely used college performance indicator is the cumulative Grade Point Average (cGPA) by virtue of its perceived value in reflecting students' in-course college performance. Generally, cGPA is considered a valid and reliable tool in university settings;^27^^, ^^28^ however, this has not been confirmed specifically for medical colleges yet. It also bears some flaws that may limit its reliability and validity, like grade inflation and institutional differences in grading.^27^ Nevertheless, to date, there has not been any better significant indicator of student performance than the cGPA, which henceforth makes it the most extensively used tool in academic research to correlate pre-admission variables with students' college performances.^27^

Another assessment method that has recently emerged as an indicator of student performance is the progress test (PT). Unlike the cGPA, the PT is a longitudinal assessment method that assesses student achievement and progression.^29^^, ^^30^ It was first introduced in Maastricht Faculty of Health in Netherlands as a tool to assess its problem-based learning (PBL) curriculum in the early 1970's.^31^^,^^32^ Since then, medical educationists have documented several advantages of the PT. For instance, the PT, which focuses on functional knowledge (knowledge that comes with repetition), provides valuable information that can be used for feedback and has research potential that can be used in education.^32^ This contributed to the development, implementation and rapid spread of various types of PTs in many medical schools worldwide; including in South Africa,^33^ Indonesia,^34^ Germany,^35^ and the US.^36^ A national PT is being administered in Saudi Arabia by the college of medicine at Qassim University and is offered to all medical colleges willing to participate.^37^

The aim of this study is to examine the predictive validity of the Saudi national pre-admission variables and English language proficiency standardized tests on medical students' performance using the PT and cGPA as indicators of student performance.

## Methods

### Participants, Setting, and Procedure

We conducted a retrospective study at the College of Medicine at Alfaisal University in Saudi Arabia, where we collected data of 737 students from all academic years through the college's Student Information System. Collected data included student demographics such as student academic year and gender; student pre-admission variables, namely: the cumulative high school average, NAT, and GAT; English proficiency test scores, namely: TOEFL and IELTS; and college academic performance indicators, namely: cGPA and PT scores.

The required ethical approval was obtained from the Institutional Review Board of Alfaisal University in Saudi Arabia. To ensure confidentiality, the data was coded by the main investigator in line with institutional ethical guidelines. Following the data coding step, statistical analysis was performed.

Descriptive analysis was performed. Multiple linear regression analyses were used to devise models that assess the predictive relationship of five pre-admission variables (namely, cumulative high school average, NAT, GAT, IELTS and TOEFL) on their cGPA and PT results, in both preclinical (years 1, 2 and 3) and clinical years (years 4, 5 and 6). All the analyses were carried out using IBM Statistical Package for the Social Sciences (SPSS) software, version 20. A p-value of <0.05 was considered significant.

## Results

Data was available for a total of 737 students. The demographic features of our study subjects are demonstrated in Table 1. The mean and standard deviation of pre-admission and college performance indicator scores are shown in Table 2.

**Table 1:** Demographic characteristics of study participants

Demographic variables		N	%
Gender	Males	409	55.5
	Females	328	44.5
Year	First	149	20.2
	Second	183	24.8
	Third	152	20.6
	Fourth	143	19.4
	Fifth	69	9.3
	Sixth	41	5.6

### Factors predicting the cGPA

Two separate models were analyzed to study the ability of the pre-admission variables to predict students' cGPA as seen in Table 3. One multiple linear regression was calculated to predict cGPA in preclinical years based on the five pre-admission variables. A significant regression equation was found (F_(__5,5)_=10.729, p=0.01), with an R^2^ of 0.951. The regression further revealed that the NAT (p=0.04, B=0.08, t=2.752) and TOEFL (p=0.017, B=0.01, t=3.527) scores were both significant positive predictors of students' cGPA in preclinical years of medical school, with the NAT being a stronger predictor. However, GAT was found to be a significant negative predictor of cGPA in the same group (p=0.048, B=-0.05, t=-2.606). Cumulative high school average and IELTS were not significant predictors of preclinical cGPA (p=0.17, p=0.691 respectively).

A second multiple linear regression was calculated to predict cGPA in clinical years based on the five pre-admission variables. This regression, on the other hand, revealed no significant predictors of cGPA among any of the five pre-admission variables included in the analysis; with an overall regression equation (F_(__5,1)_=0.654, p=0.729), and with an R^2^ of 0.766.

### Factors predicting the PT

Two separate models were analyzed to study the ability of the pre-admission variables to predict students' PT performance, as seen in Table 3.

The preclinical regression model analysis revealed no significant predictors of PT performance among any of the five pre-admission variables included in the analysis; with an overall regression equation (F_(__5,4)_=0.692, p=0.657), and with an R^2^ of 0.464.

The clinical years' model, however, did not contain sufficient data to run the analysis and is thus not included in the table. This is due to the relatively lower number of students in this phase currently at our institution.

### Prediction of PT by cGPA

Another linear regression was calculated to predict PT performance based on cGPA. A significant regression equation was found (F_(__1,387)_=59.006, p<0.0001), with an R^2^ of 0.132. The regression further revealed that the cGPA was a strongly significant predictor of students' PT performance (p<0.0001, B=10.904, t=7.682). Therefore, on average, one-point increase in cGPA predicts for almost 11 points increase in the PT.

**Table 2:** Descriptive statistics of pre-admission scores and performance indicators

Variables		Mean	SD
Pre-admission variables	CHSA	97.29	6.168
	NAT	80.55	13.562
	GAT	82.34	11.487
	TOEFL	550.92	58.930
	IELTS	6.12	0.879
Performance indicators	cGPA	3.12	0.582
	PT	22.31	15.919

CHSA= Cumulative High School Average; NAT= National Achievement Test; GAT=General Aptitude Test; TOEFL= Test of English as a Foreign Language; IELTS= International English Language Testing System; cGPA= Cumulative Grade Point Average; PT=Progress Test

## Discussion

### Academic assessment

Our findings indicate that the NAT is a significant positive predictor of student performance in preclinical years through the cGPA; however, it was not found to be predictive of performance in clinical years. This could be partially attributable to the test being focused on some of the students' academically acquired knowledge and cognitive abilities such as the ability to infer, comprehend and apply; however, it does not assess other cognitive and non-cognitive skills which influence performance in clinical years.^11^^,^^12^^,^^38^ These results are in line with other national studies conducted in Saudi Arabia. The general literature consensus is that NAT is a significant predictor of college performance in Saudi medical schools, such that in many studies it was even considered as the best predictor of the pre-admission variables.^12^^,^^39^^,^^40^

On the contrary, our results show that the cumulative high school average was not predictive of cGPA at either pre-clinical or clinical years. In the majority of studies, except Al-Rukban and colleagues,^39^ cumulative high school average was usually reported to be statistically predictive of performance in medical school.^12^^,^^40^ This difference we report could be attributed to many factors. Firstly, the cumulative high school average represents all subjects taught in high school including those that are not related to medicine.^39^ Secondly, the schools' teaching medium is mainly Arabic, while the medical college courses are exclusively conducted in English. Finally, the high school assessment system is different from that of the medical college in many ways, such as exam questions' style (multiple choice questions, short answer questions, objective structured clinical evaluation, etc.) and scope of questions (recall, communication, critical thinking, clinical application, etc.). Neither the NAT nor the CHSA was found to be predictive of performance through the PT.

**Table 3:** Pre-admission variables ability to predict cGPA and progress test in preclinical and clinical phases of the medical school

Test	Preclinical students (n=484)		Clinical students (n=253)
	cGPA B(95% CI); p-value	Progress test B(95% CI); p-value	cGPA B(95% CI); p-value
CHSA	0.21 (-0.14,0.56); 0.17	0.31 (-12.03,12.66); 0.95	-0.09 (-1.77,1.60); 0.63
GAT	**-0.05** (-0.11, -0.00); 0.048	-0.99 (-3.35,1.36); 0.31	-0.06 (-1.06,0.94); 0.57
NAT	** 0.08** (0.01,0.16); 0.04	1.05 (-1.76,3.88); 0.36	0.06 (-0.97,1.09); 0.61
TOEFL	** 0.01** (0.00,0.01); 0.02	0.10 (-0.13,0.33); 0.30	-0.00 (-0.06,0.06); 0.61
IELTS	0.12 (-0.62,0.86); 0.69	7.04 (-22.64,36.72); 0.55	0.13 (-4.69,4.94); 0.80

Note: Numbers in bold depict significant B values (p<0.05); 95% CI = 95% confidence interval. CHSA=Cumulative High School Average; NAT= National Achievement Test; GAT=General Aptitude Test; TOEFL= Test of English as a Foreign Language; IELTS= International English Language Testing System; cGPA= Cumulative Grade Point Average

### Non-academic cognitive assessments

Our findings suggest that GAT is a negative predictor of the cGPA in preclinical years in addition to not being predictive of performance during clinical years. While initially surprising, this goes hand in hand with internationally published work indicating that measures of aptitude, in general, tend not to correlate with students' university academic performance or may even negatively correlate with it.^41^ This could perhaps be attributable to several causes reported by other studies about aptitude measures, including: firstly, failure of aptitude tests in predicting the performance of students from minority groups and diverse ethnic backgrounds could sometimes be expected due to the way such tests are developed.^42^ Secondly, aptitude tests are limited to assessing cognitive variables and cease to cover non-cognitive variables which were shown to be important contributors to academic success.^42^ Studies have shown that cognitive variables constitute only around 25% of the variables affecting academic performance and success.^43^

These explanations were reported to apply to general aptitude measures, and since GAT is an aptitude test, we hypothesize that these might provide a possible explanation to our findings; however, more studies are needed to explore this area further.

In comparison, several studies have been conducted in Saudi Arabia to assess the predictive validity of GAT on the cGPA with mixed outcomes. Some of these studies reported that GAT is predictive of performance at different levels of medical school.^12^^,^^44^ Whereas other studies, namely those conducted by Al-Rukban and colleagues, and Murshid reported that GAT is not predictive of the cGPA.^39^^, ^^45^

Moreover, our results show that GAT, similarly to the academic achievement assessments, also does not predict performance through PT.

### English language proficiency assessment

Our results found the TOEFL scores to be predictive of cGPA in preclinical years. However, this was not observed in clinical years. There are several possible explanations for this observation. Firstly, during the preclinical phase, the inadequate English proficiency of non-native speakers would significantly impact their performance as it would be strongly reflected in their studies and assessment, which goes in hand with the reported predictability of TOEFL on PBL performance.^46^ Whereas by the time the students are in their clinical years, their English proficiency would improve gradually as they would have had already got used to studying and conversing in an English medium for the period of their preclinical phase.

Secondly, the TOEFL was found to be predictive of preclinical performance possibly due to the fact that the test mainly involves components that require reading and writing skills, which are strongly reflected in preclinical years' assessment. This is in contrast to clinical years that focus more on communication skills and require more verbal proficiency, both of which are not adequately reflected by the test. This limitation in verbal proficiency and communication skills assessment is supported in Roemer's study which highlights that the TOEFL's prediction accuracy is limited to only grammar and reading proficiency.^47^ This also goes in hand with the recommendations of the Educational Testing Service (ETS) to not use rigid cut-off TOEFL scores for student selection.^48^

In contrary, IELTS was not found to be predictive of performance in either preclinical or clinical years. Neither the TOEFL nor the IELTS was found to be predictive of performance through the PT.

### College performance indicators: cGPA and PT

Our results show a positive correlation with strong predictive validity. Giving the several additional advantages mentioned earlier for the PT over the cGPA, namely functional knowledge; being able to quantify the cGPA's predictability of the PT provides educators with a valuable tool to better monitor students' progress and performance over time, permitting a more detailed scope for analysis of students' annual performance in specific subject disciplines. This valuable information could be used to improve and inform mentorship programs such that students would be able to receive individualized feedback on their annual performance progression, in a detailed and subject-wise manner.^49^

Despite the wide use of PT and cGPA, the literature investigating the relationship between them is quite deficient. Our literature search identified only one study by Al Alwan and colleagues that touched upon this relation,^29^ such that a positive correlation between the PT and cGPA was indicated; however, the predictive validity of this correlation was not reported. Our study offers the advantage of bridging this literature gap through quantifying this predictive validity.

### Pre-admission variables and PT

While our results show some of the pre-admission variables to be predictive of performance through the cGPA, none of them succeeded in predicting pre-clinical students' performance through the PT. This is mostly attributable to the fact that the predictive validity of the pre-admission variables does not usually include examining functional knowledge. Therefore, upon adding the PT data to our study analysis, pre-admission variables that previously demonstrated to be predictive of student performance with the cGPA appeared to lose their performance predictability.

### Limitations

Since we are a relatively new school, the sample size of our students currently in the clinical years was not sufficient to examine the predictability of pre-admission variables on PT in that phase, but as more of our students' progress into the clinical years, we will be able to explore this area further. Another applicable limitation is that the study represents data of a single school in Saudi Arabia. Thus, further studies using data from multiple schools would be needed to validate these findings further.

## Conclusions

Our study shows that among all the pre-admission variables currently used in Saudi Arabia, the NAT and TOEFL scores are the only positive predictors of performance in the preclinical years when reflected by students' cGPA. However, when using the PT as a performance indicator, the predictive effects of those variables cease; and it is likely so due to the additional assessment of the longitudinal functional knowledge component by the PT. We henceforth recommend using the PT, in addition to the cGPA, when examining the predictive validity of pre-admission variables at other institutions in Saudi Arabia and around the world. Lastly, we believe that there is a pressing need to recognize or develop more sensitive pre-admission assessment variables that can better predict student performance in the clinical years, and that much further research in this area is yet anticipated to underpin the existing ones.

### Acknowledgements

The authors sincerely thank Dr. Mohammed Saleh Alowayed, former Dean of Admission & Registration in the Student Affairs Department at Alfaisal University, for his gracious efforts in facilitating the data collection process from the Student Information System. The authors extend the acknowledgement to Prof. Khaled Al-Kattan, Dean of College of Medicine, Alfaisal University and Dr. Wael Al-Kattan, Vice Dean for Academic and Student Affairs, College of Medicine, Alfaisal University for their continuous care and unlimited support to this study.

### Conflict of Interest

The authors declare that they have no conflict of interest.
